# Low Back Pain Among Employees of the Qassim Health Cluster: Prevalence, Patterns, and Predictive Factors

**DOI:** 10.7759/cureus.103469

**Published:** 2026-02-12

**Authors:** Asim Abdelgader Farah, Abdulmajeed A Alateeq, Rayan Mohammed M Alismail, Nihal Abdelrazig Mohammed

**Affiliations:** 1 Department of Orthopedics, Qassim Health Cluster, King Fahad Specialist Hospital, Buraydah, SAU; 2 Department of Diabetology, Diabetes and Endocrine Centre, Qassim Health Cluster, King Fahad Specialist Hospital, Buraydah, SAU

**Keywords:** disability, healthcare, low back pain, pain management, saudi arabia

## Abstract

Background: The purpose of this study is to examine the prevalence of low back pain (LBP) and its association with demographic and occupational factors and disability among healthcare professionals and administrative staff in the Qassim Health Cluster in Saudi Arabia.

Methods: A cross-sectional study was employed using a self-administered questionnaire. Data collection was carried out from 323 workers in Qassim Health Cluster, Saudi Arabia. The questionnaire gathered data based on demographics, lifestyle, occupational factors, LBP characteristics, the Visual Analog Scale (VAS) for pain, and the Oswestry Disability Index (ODI). Data were analyzed using descriptive statistics, chi-square tests, t-tests, ANOVA, and multiple linear regression.

Results: LBP prevalence was high among nurses and doctors belonging to the age group 31-50 years. The significant factors associated with LBP and ODI scores are lack of regular exercise, inadequate sleep, lifting heavy weight, high pain, and being of the female gender. Multiple linear regression depicted that current pain VAS (β = 0.38, p < 0.001), pain radiating to the legs (β = 0.26, p < 0.001), and daily sleeping hours (β = −0.11, p = 0.019) were independent predictors of disability, explaining 36.9% of the variance in ODI.

Conclusion: LBP prevalence is high (84.8%) among workers in the Qassim Health Cluster, with significant functional disability. There is a need to plan interventions in order to improve workers’ health and to engage in a work-life balance approach to overcome LBP and enhance productivity.

## Introduction

Low back pain (LBP) is related to any self-reported pain or discomfort in the body between the inferior gluteal folds and the costal margins and is a leading cause of work-related disability worldwide, particularly in occupations involving prolonged standing, lifting, and repetitive tasks. Hence, it is the most commonly reported symptom across the globe [1]. It is also a leading cause of disability worldwide, affecting 570 million people [2,3]. Most patients look for treatment as LBP disrupts their routine life and limits their ability to perform their routine tasks. Moreover, LBP is reported among working professionals. Around 39% of the adult population is affected by LBP [4]. LBP treatment is quite expensive, especially in the United States, with spending around $134.5 billion per year by healthcare centers between 1996 and 2016 [5]. Management conducts orientation sessions focused on reducing LBP and providing awareness on staying active, resting, and self-care [6]. Thus, the nonspecific LBP is the most common type faced by working individuals [7]. The prevalence of LBP over a period of one year appears in adults and gradually increases to the mid-age period [8]. However, LBP increases the disability over time and results in less engagement in everyday activities [9].

Although several studies have examined LBP in the Saudi population, the frequency, risk factors, and disability assessed using the Oswestry Disability Index (ODI) among healthcare professionals remain underexplored. Thus, most of the research findings are taken from work associated with musculoskeletal conditions [10]. Some of the studies have covered environmental risk factors, such as heavy weightlifting, standing, sitting, and job satisfaction, associated with LBP [11,12]. However, there is limited evidence of ODI, with the prevalence of LBP focusing on healthcare professionals in Saudi Arabia.

There is a scarcity of scholarly work in the context of healthcare professionals regarding the prevalence and disability index of LBP. To the best of our knowledge, there have been few focused studies on LBP and disability under cross-sectional study in healthcare professionals in Saudi Arabia. Therefore, this study examines the 12-month prevalence of LBP and its association with demographic and occupational factors and disability among healthcare professionals and administrative staff.

Literature review

Saudi Arabia has experienced public health challenges, which have led to extensive reforms [13,14]. The prevalence rate is higher in the high-income countries (30%) than in low-income countries (18.2%), and it is higher among females than among males [7]. Therefore, it is necessary to take necessary actions in order to deal with public health issues, considering the serious challenges to the healthcare system [7,9]. A recent study indicated that LBP is highly prevalent among younger age groups [16].

LBP causes a loss of productive days among workers; in the United Kingdom, it is 116 million days, which costs 12 billion pounds annually [17]. In Europe, the cost is estimated at 7000 euros [18] and 740 euros in Sweden [19], per person annually. Moreover, LBP leaves a negative financial impact due to less efficient health systems and a lack of resources to deal with these challenges. Several studies have indicated risk factors linked to LBP, such as aging, drugs, gender, obesity, smoking, back injury, long-standing, and sitting [20,21]. Keeping in view the risk factors associated with LBP helps to design policies and strategies to cope with the fundamental issues. However, healthcare workers are the most affected group and need utmost attention to set a better health system for them [8]. Specifically, hospital workers show a high rate of LBP as compared to the general population, which indicates their higher engagement with work and depicts high stress in their work [22].

Research studies have mentioned that conditions such as work shifts, overtime work, and working posture are among the most important predictors of LBP [23,24]. Moreover, lifestyle indicators such as psychological issues, mainly job stress, obesity, and less physical activity could be the reason for LBP [25,26]. Specifically, a study identified that 44% of nurses sought medical advice and 8.6% were hospitalized due to LBP in Jordan [26]. A systematic review of 154 studies mentioned a pooled lifetime LBP prevalence of 54.8% among healthcare workers and associated factors, mainly body mass, work-related factors, and gender [27]. Another review study of nurses in Africa indicated a 64.1% LBP prevalence [28]. Another systematic review study related to the prevalence of LBP in Saudi Arabia mentioned that the working group has LBP prevalence between 64% and 89%, which is most common among female workers [10].

Hypothesis and justification

The study hypotheses are developed based on the LBP prevalence over 12 months and linked to disability among workers at Qassim Health Cluster, who are affected by lifestyle, occupational, and clinical factors.

H1: LBP prevalence over 12 months is high among employees of Qassim Health Cluster, particularly among healthcare professionals (nurses and doctors), compared to administrative staff. H2: Employees with fewer daily sleeping hours (<6 hours) have higher ODI scores than those with longer sleep duration. H3: Employees who exercise regularly have lower LBP prevalence and ODI scores. H4: Employees who lift heavy weights or equipment have higher ODI scores than those who do not. H5: Higher current pain intensity (VAS score) predicts higher ODI scores. H6: Pain radiating to the legs predicts higher ODI scores. H7: Nurses and doctors have a higher prevalence of LBP than administrative staff and other employees. H8: Smoking or ex-smoking employees have higher ODI scores than non-smokers. H9: Longer sitting or standing hours during work are associated with higher ODI scores.

Study objective

The primary objective of the study is to determine the 12-month prevalence of LBP among employees of the Qassim Health Cluster in Saudi Arabia. The secondary objectives of the study are to describe clinical patterns of LBP (e.g., severity, chronicity, associated symptoms) and to identify predictive factors associated with LBP (e.g., job role, BMI, ergonomics, physical activity, stress).

## Materials and methods

An analytical cross-sectional study design was employed using a self-administered questionnaire. The target population was workers, including healthcare professionals and administrative staff from the Qassim Health Cluster in Saudi Arabia. The study participants included doctors, nurses, technicians, administrative staff, and other health professionals.

Individuals who were not working in Qassim Health Cluster healthcare facilities were excluded from the study. Moreover, employees who were on medical leave or diagnosed with serious spinal pathology (e.g., spinal tumors, infections) and temporary staff or interns were also excluded.

Data collection was carried out from October to November 2025 via an electronic and paper-based questionnaire. The English version of the questionnaire was used for data collection.

A questionnaire comprised several parts, such as demographics (age, gender, role, years of experience), lifestyle factors (smoking, physical activity, sleep), work-related factors (sitting hours, lifting, stress level), LBP characteristics (duration, intensity, recurrence), the Visual Analog Scale (VAS) for pain, and the ODI index as validated by Fairbank & Pynsent [29].

According to the WHO manual, the sample size calculation with an estimate of 45% of LBP with a precision of 95% confidence interval [30,31] had a minimal sample size of 323 healthcare professionals in the Qassim Health Cluster. A convenience sampling technique was used due to access and time constraints, allowing recruitment during working hours to gather responses from consenting participants. Hence, this approach limits external validity, and study findings should be generalized beyond the study population with caution.

Ethical approval was obtained from the IRB of the Qassim Health Cluster in Saudi Arabia

Data analysis was performed with IBM Corp. Released 2021. IBM SPSS Statistics for Windows, Version 27. Armonk, NY: IBM Corp. Responses with missing data were dealt with using listwise deletion. Descriptive statistics were depicted as frequency and percentage. A comparison between LBP presence and absence across demographic and occupational groups was conducted. Further, differences in ODI for two-group factors using an independent t-test and multi-group factors using ANOVA were conducted. Before inferential analyses, normality was checked using Q-Q plots; homogeneity of variances was assessed with Levene’s test for ANOVA and t-tests. For regression, residual diagnostics were examined to assess the normality of residuals.

## Results

Profile of study participants

The response rate was 68.2% (n=341) of the questionnaires received out of 500 distributed. After data cleaning and screening, 323 responses were included in the analysis. Table 1 presents the characteristics of study participants, where most participants were from the age group 31-40 years (42.7%), belonged to the nursing (33.1%) or doctor (32.2%) profession, sat for 4-8 hours at work (48.6%), lifted heavy weights (35.6%), were non-smokers (74.6%), slept for 6 to 8 hours daily (63.5%), and were not engaged in exercise (79.3%).

**Table 1:** Participant profile (n=323)

Variables	Category	n (%)
Age (years)	18–30	33 (10.2)
	31–40	138 (42.7)
	41–50	114 (35.3)
	51–60	35 (10.8)
	>60	3 (0.9)
Gender	Female	111 (34.4)
	Male	212 (65.6)
Height (cm)	<160	73 (22.6)
	161–170	127 (39.3)
	171–180	92 (28.5)
	181–190	31 (9.6)
Weight (kg)	<50	4 (1.2)
	51–60	34 (10.5)
	61–70	72 (22.3)
	71–80	86 (26.6)
	81–90	66 (20.4)
	91–100	30 (9.3)
	>100	31 (9.6)
Occupation	Administrative staff	39 (12.1)
	Doctor	104 (32.2)
	Nurse	107 (33.1)
	Technician	51 (15.8)
	Other health professions	22 (6.8)
Sitting hours at work	<4 hours	136 (42.1)
	4–8 hours	157 (48.6)
	>8 hours	30 (9.3)
Standing hours at work	<4 hours	152 (47.1)
	4–8 hours	122 (37.8)
	>8 hours	49 (15.2)
Lifting heavy weights	No	208 (64.4)
	Yes	115 (35.6)
Smoking status	No	241 (74.6)
	Yes	57 (17.6)
	Ex-smoker	25 (7.7)
Sleep duration (hours/day)	<6	106 (32.8)
	6–8	205 (63.5)
	9–10	10 (3.1)
	>10	2 (0.6)
Regular exercise	No	256 (79.3)
	Yes	67 (20.7)

Prevalence of LBP

Table 2 depicts the prevalence of LBP with demographic, lifestyle, and occupational characteristics. Significant differences were reported for LBP prevalence for age (p = 0.003), occupation (p = 0.005), regular exercise (p < 0.001), current pain intensity (VAS; p < 0.001), pain radiating to the legs (p = 0.002), sick leave due to pain (p = 0.050), and ODI disability level (p = 0.002). Overall LBP prevalence among all ages is 84.8. LBP prevalence was high for age group 31-50 years, nurses and doctors, those not engaged in exercise, those reporting moderate to severe pain, and those with radiating pain or higher disability levels. No significant associations were identified for gender, sitting or standing duration, height, weight, lifting heavy weights, sleep duration, and smoking status (all p > 0.05).

**Table 2:** Prevalence of LBP with occupational and demographic of participants LBP: Low back pain

Variable	Category	LBP-No n (%)	LBP-Yes n (%)	Prevalence LBP (%)	χ² (df)	p-value (Sig. at < 0.05)
Age	18–30	11 (33.3)	22 (66.7)	6.8	15.825 (4)	0.003
	31–40	17 (12.3)	121 (87.7)	37.5		
	41–50	11 (9.6)	103 (90.4)	31.9		
	51–60	9 (25.7)	26 (74.3)	8.0		
	>60	1 (33.3)	2 (66.7)	0.6		
	Total among all ages	49 (15.2)	274 (84.8)	84.8		
Gender	Female	14 (12.6)	97 (87.4)	30.0	0.860 (1)	0.354
	Male	35 (16.5)	177 (83.5)	54.8		
Height (cm)	<160	10 (13.7)	63 (86.3)	19.5	4.200 (3)	0.241
	161–170	18 (14.2)	109 (85.8)	33.7		
	171–180	19 (20.7)	73 (79.3)	22.6		
	181–190	2 (6.5)	29 (93.5)	9.0		
Weight (kg)	<50	1 (25.0)	3 (75.0)	0.9	1.242 (6)	0.975
	51–60	6 (17.6)	28 (82.4)	8.7		
	61–70	11 (15.3)	61 (84.7)	18.9		
	71–80	13 (15.1)	73 (84.9)	22.6		
	81–90	10 (15.2)	56 (84.8)	17.3		
	91–100	5 (16.7)	25 (83.3)	7.7		
	>100	3 (9.7)	28 (90.3)	8.7		
Occupations	Administrative Staff	2 (5.1)	37 (94.9)	11.5	30.064 (13)	0.005
	Doctor	18 (17.3)	86 (82.7)	26.6		
	Nurse	13 (12.1)	94 (87.9)	29.1		
	Technician	9 (17.6)	42 (82.4)	13.0		
	Other	7 (31.8)	15 (68.2)	4.6		
Sitting hours during work	<4 hours	23 (16.9)	113 (83.1)	35.0	0.977 (2)	0.614
	4–8 hours	23 (14.6)	134 (85.4)	41.5		
	>8 hours	3 (10.0)	27 (90.0)	8.4		
Standing hours during work	<4 hours	21 (13.8)	131 (86.2)	40.6	2.441 (2)	0.295
	4–8 hours	23 (18.9)	99 (81.1)	30.7		
	>8 hours	5 (10.2)	44 (89.8)	13.6		
Lifting heavy weights/equipment	No	32 (15.4)	176 (84.6)	54.5	0.021 (1)	0.885
	Yes	17 (14.8)	98 (85.2)	30.3		
Smoking	No	35 (14.5)	206 (85.5)	63.8	3.646 (2)	0.162
	Yes	7 (12.3)	50 (87.7)	15.5		
	Ex-smoker	7 (28.0)	18 (72.0)	5.6		
Sleep hours (daily)	<6 hours	10 (9.4)	96 (90.6)	29.7	5.687 (3)	0.128
	6–8 hours	36 (17.6)	169 (82.4)	52.3		
	9–10 hours	2 (20.0)	8 (80.0)	2.5		
	>10 hours	1 (50.0)	1 (50.0)	0.3		
Exercise regularly	No	29 (11.3)	227 (88.7)	70.3	14.157 (1)	<0.001
	Yes	20 (29.9)	47 (70.1)	14.5		
Current pain VAS	No pain (0)	12 (57.1)	9 (42.9)	2.8	57.661 (3)	<0.001
	Mild pain (1–3)	9 (9.0)	91 (91.0)	28.2		
	Moderate pain (4–6)	1 (0.8)	124 (99.2)	38.4		
	Severe pain (7–10)	0 (0.0)	50 (100.0)	15.5		
Pain radiating to legs	No	22 (12.5)	154 (87.5)	47.7	9.340 (1)	0.002
	Yes	3 (2.5)	118 (97.5)	36.5		
Sick leave because of pain	No	29 (11.9)	215 (88.1)	66.6	3.846 (1)	0.050
	Yes	2 (3.3)	58 (96.7)	18.0		
ODI disability level	Minimal disability	47 (20.3)	185 (79.7)	57.3	16.635 (4)	0.002
	Moderate disability	2 (2.6)	74 (97.4)	22.9		
	Severe disability	0 (0.0)	12 (100.0)	3.7		
	Crippled	0 (0.0)	2 (100.0)	0.6		
	Bed-bound / Exaggerating symptoms	0 (0.0)	1 (100.0)	0.3		

Group comparison with ODI scores

Table 3 shows differences in ODI scores with two group factors using an independent t-test. Significant differences were noticed in the females as compared to males in terms of ODI score (p = 0.002), and in workers who lifted heavy weights compared to those who did not (p = 0.035). No significant differences were identified for sitting hours, exercise status, standing hours, and smoking status (all p > 0.05).

**Table 3:** Differences in ODI with two-group factors using independent t-test

Variable	Group	N	Mean ± SD	t (df)	p-value (Sig. at < 0.05)	Mean Difference (95% CI)	Cohen’s d
Gender	Male	212	13.27 ± 13.51	-3.17 (321)	0.002	-5.07 (-8.22, -1.92)	-0.37
	Female	111	18.34 ± 13.94				
Exercise regularly	Yes	67	15.22 ± 18.46	0.14 (321)	0.890	0.26 (-3.48, 4.01)	0.02
	No	256	14.96 ± 12.41				
Lift heavy weights/equipment	Yes	115	17.20 ± 13.53	2.12 (321)	0.035	3.39 (0.24, 6.54)	0.25
	No	208	13.81 ± 13.91				
Sitting hours during work	≥4 hours	187	15.39 ± 14.92	0.57 (321)	0.569	0.89 (-2.18, 3.96)	0.06
	<4 hours	136	14.50 ± 12.27				
Standing hours during work	≥4 hours	171	15.39 ± 14.77	0.52 (321)	0.605	0.80 (-2.24, 3.84)	0.06
	<4 hours	152	14.59 ± 12.78				
Smoking	Smoker/Ex-smoker	82	14.37 ± 12.91	-0.49 (321)	0.624	-0.87 (-4.36, 2.62)	-0.06
	Non-smoker	241	15.24 ± 14.18				

Table 4 depicts differences in ODI with multi-group factors using ANOVA. There were no significant differences by age groups, height, and weight (all p > 0.05). Comparatively, a significant association was reported between ODI and sleep hours per day (F = 4.01, p = 0.008) in those who sleep less than 6 hours per day as compared to those who take long sleep.

**Table 4:** Differences in ODI with multi-group factors using ANOVA

Variable	Category	N	Mean ± SD	F (df)	p-value (Sig. at < 0.05)
Age (years)	18–30	33	9.27 ± 13.16	2.38 (4)	0.052
	31–40	138	16.06 ± 13.68		
	41–50	114	15.56 ± 11.89		
	51–60	35	15.66 ± 19.42		
	>60	3	2.00 ± 2.00		
Height (cm)	<160	73	16.66 ± 15.06	1.19 (3)	0.313
	161–170	127	15.83 ± 14.58		
	171–180	92	13.15 ± 12.21		
	181–190	31	13.35 ± 12.16		
Weight (kg)	<50	4	3.00 ± 4.76	0.95 (6)	0.460
	51–60	34	14.76 ± 14.69		
	61–70	72	14.78 ± 12.47		
	71–80	86	16.33 ± 14.76		
	81–90	66	15.73 ± 12.03		
	91–100	30	11.73 ± 18.32		
	>100	31	15.42 ± 12.65		
Sleep hours (daily)	<6 hours	106	18.47 ± 14.42	4.01 (3)	0.008
	6–8 hours	205	13.45 ± 13.44		
	9–10 hours	10	13.40 ± 9.80		
	>10 hours	2	0.00 ± 0.00		

Factors influencing ODI scores

Table 5 depicts a multiple linear regression analysis determining factors associated with ODI. Multicollinearity was checked using the Variance Inflation Factor (VIF); all values were below 5, indicating no multicollinearity. It shows a significant association with current pain VAS (β = 0.38, p < 0.001), pain radiating to the legs (β = 0.26, p < 0.001), and daily sleeping hours (β = −0.11, p = 0.019). The model explained 36.9% of the variance in ODI and was statistically significant (F(7, 284) = 23.72, p < 0.001).

**Table 5:** Multiple linear regression.

Predictor	β	t	p-value (Sig. at < 0.05)
Age	0.03	0.52	0.604
Smoking	−0.04	−0.88	0.378
Daily sleeping hours	−0.11	−2.36	0.019
Exercise regularly	0.08	1.71	0.089
Current pain VAS score	0.38	7.50	<0.001
Pain radiating to legs	0.26	5.08	<0.001
Model statistics			
R² / Adjusted R²			0.369 / 0.353
F (df)			23.72 (7, 284)
Model p-value			<0.001
Dependent variable: Oswestry Disability Index (ODI) score (%).			

Table 6 depicts summary of hypotheses.

**Table 6:** Summary of hypotheses

No	Hypothesis Statement	Type	Statistical Support	Notes	Source Table
H1	LBP prevalence is high among employees of Qassim Health Cluster, particularly among healthcare professionals (nurses and doctors) compared to administrative staff.	Supported	χ² p = 0.005	Prevalence highest among nurses (87.9%) and doctors (82.7%)	Table 2
H2	Employees with fewer daily sleeping hours (<6 hours) have higher ODI scores than those with longer sleep duration.	Supported	ANOVA p = 0.008, regression β = −0.11, p = 0.019	Negative association between sleep hours and disability	Tables 4 & 5
H3	Employees who exercise regularly have lower LBP prevalence and ODI scores.	Exploratory	χ² p < 0.001 for prevalence, regression p = 0.089	Significant for prevalence, not significant in regression	Tables 2 & 5
H4	Employees who lift heavy weights or equipment have higher ODI scores than those who do not.	Supported	t-test p = 0.035, Cohen’s d = 0.25	Significant predictor	Table 3
H5	Higher current pain intensity (VAS score) predicts higher ODI scores.	Supported	Regression β = 0.38, p < 0.001	Strong predictor of disability	Table 5
H6	Pain radiating to the legs predicts higher ODI scores.	Supported	Regression β = 0.26, p < 0.001	Significant predictor	Table 5
H7	Nurses and doctors have higher prevalence of LBP than administrative staff and other employees.	Supported	χ² p = 0.005	Descriptive support from prevalence data	Table 2
H8	Smoking or ex-smoking employees have higher ODI scores than non-smokers.	Exploratory	Regression p = 0.378	Not significant in regression	Table 5
H9	Longer sitting or standing hours during work are associated with higher ODI scores.	Exploratory	Regression p > 0.05	Not supported	Tables 3 & 5

## Discussion

This study examined the 12-month LBP prevalence, risk factors, and disability among healthcare and administrative workers in Qassim Health Cluster, Saudi Arabia. The study identified high LBP prevalence among doctors, nurses, and people with disabilities for several demographic, occupational, and lifestyle factors. Similar to earlier studies, LBP prevalence was a high rate among healthcare workers, as it is linked to physical tasks such as standing and repetitive movements [1]. In this study, high prevalence was noted among the age group 31-50 years, as this is aligned with evidence that the age factor is linked to musculoskeletal degeneration and increased risk for LBP [32].

Most importantly, lifestyle plays a significant role, as those participants who are not engaged in regular exercise or physical activity showed high prevalence and disability due to LBP. Less physical activity is linked to poor muscle strength, which can lead to musculoskeletal disorders and LBP [5]. Moreover, inadequate sleep of less than six hours increased the ODI score. Additionally, lifting heavy weights was significantly linked to higher disability.

It urges the importance of interventions to address LBP and disability among healthcare workers. Thus, factors such as variability in posture, supportive tools while working, and work breaks could overcome the LBP in healthcare settings [10]. Pain severity also appeared as a significant factor for disability, as those who reported moderate to severe pain, radiating leg pain, and sick leave history because of LBP showed high ODI scores. The linear regression analysis reported that current pain intensity (VAS score), sick leave history, radiating pain, and sleep duration were independently related to disability, explaining 36.9% of the variance in ODI scores. In terms of gender, females showed a higher ODI score compared to males. Hence, there is a need to examine two factors in detail linked to LBP and disability among females.

Research studies report that the one-year prevalence can provide a more accurate number of prevalence, as this reduces bias related to the population in the study [33]. LBP prevalence numbers may change from country to country. LBP prevalence among nurses in Taiwan was reported as 72% [34]. In Turkey, 53% of LBP was reported among healthcare workers [35]. In another study, a Turkish hospital reported LBP prevalence among nurses (77.1%), physicians (63.3%), and physical therapists (72.7%) [36]. In the southwest part of Saudi Arabia, LBP prevalence accounted for 73.9% in the past 12 months [37]. Hence, several studies indicated that personal and work-related factors lead to LBP among healthcare workers [38-40]. LBP prevalence differs across different studies and is difficult to generalize. Thus, changes in LBP prevalence among different groups, regions, and countries are due to personal criteria and working conditions [32].

This study took advantage of the assessment of multiple groups in terms of LBP prevalence. A multifactorial analysis was conducted based on the occupation and lifestyle of the study participants, including exercise, sitting and standing while working, sleeping hours, smoking, and weightlifting. Moreover, the VAS pain scale and ODI index were used to ensure the reliability and comparability of study findings. Furthermore, the large sample size gives an edge to analyze the data with sufficient statistical power and assessment of risk factors that could support preventive interventions.

Keeping in view the study’s strengths, this study has some limitations in terms of study design, sample, and generalizability. The cross-sectional design limits the causal relation between factors and LBP. Self-reporting bias may occur due to data reported for exercise, sleep, and pain. Moreover, some of the subgroups in the category of age, gender, and occupational indicators had a small sample size. This study was limited in the clinical assessment of pain, which could minimize the accuracy of pain diagnosis. Finally, the sample size is limited to the Qassim Health Cluster; generalizability to other sets of populations may be limited.

The study findings relate to several recommendations in terms of workplace intervention, longitudinal study, and assessment of high-risk LBP groups. Workplace intervention could be planned to motivate working professionals to opt for regular exercise and adequate sleep to overcome the LBP risks. A longitudinal study can be conducted for better assessment of risks related to LBP over time. Preventive programs can be conducted with doctors, nurses, and administrative staff to have a better work-life balance. Furthermore, awareness campaigns and education for workers in the Qassim Health Cluster about reporting of LBP, timely treatment, correct posture, and weightlifting techniques could be supportive to reduce LBP and address challenges related to disability.

## Conclusions

The purpose of this study is to enhance employee productivity by addressing and overcoming the underlying causes of low back pain. LBP prevalence over 12 months is high among healthcare workers in Qassim Health Cluster, Saudi Arabia, as it shows that affected individuals are mostly nurses and doctors in the age group 31-50 years. Disability is linked to factors such as pain intensity, pain radiating to the legs, sick leave history because of pain, lack of sleep, less physical activity, and female gender. Moreover, an individual who lifts heavy weights is more likely to have higher disability. These findings urge the need to launch interventions that provide awareness and guidance to cope with LBP on time. Moreover, this study calls for the launch of education and management programs for healthcare workers.

## Appendices

Questionnaire

Section A: Demographic Information

Q1. Age ☐ 18-30 ☐ 31-40 ☐ 41-50 ☐ 51-60 ☐ >60

Q2. Sex ☐ Male ☐ Female

Q3. Height (cm) ☐ <160 ☐ 161-170 ☐ 171-180 ☐ 181-190 ☐ >190

Q4. Weight (kg) ☐ <50 ☐ 51-60 ☐ 61-70 ☐ 71-80 ☐ 81-90 ☐ 91-100 ☐ >100

Q5. Occupation ☐ Doctor ☐ Nurse ☐ Technician ☐ Administrative Staff ☐ Other: __________

Section B: Work Style

Q6. Sitting Hours During Work ☐ Less than 4 hours ☐ 4-8 hours ☐ More than 8 hours

Q7. Standing Hours During Work ☐ Less than 4 hours ☐ 4-8 hours ☐ More than 8 hours

Q8. Do you lift heavy weights or equipment at work? ☐ Yes ☐ No

Section C: Lifestyle & Habits

Q9. Do you smoke? ☐ Yes ☐ No ☐ Ex-smoker

Q10. Daily Sleeping Hours ☐ Less than 6 hours ☐ 6-8 hours ☐ 9-10 hours ☐ More than 10 hours

Q11. Do you exercise regularly? ☐ Yes ☐ No

Section D: Low Back Pain - Past 12 Months

Q12. Have you had lower back pain in the past year? ☐ Yes ☐ No

Q13. If yes, how long did the pain last most recently? ☐ Less than 1 week ☐ 2 weeks to 1 month ☐ More than 1 month

Q14. Current Pain Intensity (VAS Scale: 0-10) ☐ 0 ☐ 1 ☐ 2 ☐ 3 ☐ 4 ☐ 5 ☐ 6 ☐ 7 ☐ 8 ☐ 9 ☐ 10

Q15. Does the pain radiate to your legs? ☐ Yes ☐ No

Q16. Have you taken sick leave because of low back pain? ☐ Yes ☐ No

Section E: Oswestry Low Back Pain Disability Index

Q17. Pain Severity ☐ No pain at the moment ☐ Slight pain ☐ Moderate pain ☐ Severe pain ☐ Very severe pain ☐ Worst pain imaginable

Q18. Personal Care (e.g., bathing, dressing) ☐ No difficulty ☐ Careful but independent ☐ Sometimes need help ☐ Need daily help ☐ Need a lot of help ☐ Completely dependent

Q19. Lifting ☐ Lift anything without pain ☐ Lift heavy items with pain ☐ Lift heavy items only if close ☐ Lift only light objects ☐ Cannot lift from the ground ☐ Cannot lift at all

Q20. Walking ☐ Walk any distance ☐ Pain after long walks ☐ Limited to <1 km ☐ Limited to <500 m ☐ Need walking aid ☐ Cannot walk

Q21. Sitting ☐ No restriction ☐ Sit >1 hour with pain ☐ Sit <1 hour ☐ Sit only 30 minutes ☐ Sit <10 minutes ☐ Cannot sit

Q22. Standing ☐ Stand without limit ☐ Stand >1 hour ☐ Stand 30 minutes ☐ Stand 10 minutes ☐ Need support ☐ Cannot stand

Q23. Sleeping ☐ No effect on sleep ☐ Slight effect ☐ Difficulty sleeping ☐ Wake sometimes ☐ Wake frequently ☐ Cannot sleep

Q24. Social Life ☐ Full participation ☐ Most activities ☐ Some activities ☐ Few activities ☐ Household only ☐ None

Q25. Traveling ☐ No difficulty ☐ Some pain ☐ Uncomfortable but possible

☐ Difficult, frequent breaks ☐ Short distances only ☐ Cannot travel

Q26. Sexual Activity (Married participants only) ☐ Normal, no pain ☐ Normal with some pain ☐ Very painful ☐ Severely restricted ☐ Nearly absent ☐ No sexual activity due to pain
